# SpaVGN: A hybrid deep learning framework for high-resolution spatial transcriptomics data reconstruction and spatial domain identification

**DOI:** 10.1371/journal.pone.0329122

**Published:** 2025-08-14

**Authors:** Haiyan Wang, Yanping Zhang, Yangyang Zhang, Xuening Zhao, Zijia Bai, Xuejing Ma, Chunguang Zhao

**Affiliations:** 1 School of Mathematics and Physics, Hebei University of Engineering, Handan, China; 2 School of Mathematics and Physics, Handan University, Handan, China; Bayer Crop Science United States: Bayer CropScience LP, UNITED STATES OF AMERICA

## Abstract

Spatial transcriptomics has revolutionized the analysis of gene expression while preserving tissue spatial information, which provides novel insights into the cellular composition and function of complex biological tissues. However, current technologies are constrained by limited resolution and data sparsity, compromising the accuracy of downstream analyses. To address these challenges, we developed SpaVGN, a deep learning framework integrating convolutional neural networks, vision transformer, and graph neural networks for high-fidelity gene expression imputation and spatial domain identification. By combining local feature extraction, global attention mechanisms, and spatial graph-based modeling, SpaVGN effectively reconstructs missing transcriptomic data while preserving spatial tissue architecture. Evaluated on melanoma and sagittal posterior mouse brain datasets, SpaVGN outperformed existing methods in gene expression prediction, achieving Pearson correlation coefficients of 0.609 (melanoma) and 0.682 (mouse brain). It clearly delineated tumor regions and lymphoid niches in melanoma tissue, achieving fine-grained resolution of hippocampal subfields, including Cornu Ammonis and Dentate Gyrus, with a Silhouette Score of 0.43 and a Davies-Bouldin Index of 0.86. Validation through UMAP dimensionality reduction and PAGA network analysis demonstrated that SpaVGN significantly mitigates the negative impact of data sparsity in spatial transcriptomics, improving data completeness and spatial continuity. This study presents an innovative solution that enhances the resolution of spatial transcriptomics data, offering cross-tissue applicability and providing a valuable tool for research in biological development, disease, and tumor heterogeneity.

## 1. Introduction

Spatial transcriptomic technologies enable the analysis of gene expression while preserving the spatial architecture of tissues—an essential feature for decoding the organization and function of complex cellular environments [1–3]. These technologies fall broadly into two main categories based on data acquisition: imaging-based technologies and sequencing-based technologies. The former includes methods such as MERFISH [4], seqFISH [5], and osmFISH [6], while sequencing-based methods include 10x Visium [7], Slide-seqV2 [8], Stereo-seq [9], and DBiT-seq [10]. Imaging-based methods detect transcripts through in situ sequencing or hybridization-based capture probes, offering subcellular resolution but limited throughput and transcriptome coverage [11]. In contrast, sequencing-based approaches rely on next-generation sequencing (NGS) technologies that associate transcripts with encoded spatial coordinates prior to sequencing [12,13], enabling high-throughput and unbiased coverage for whole-transcriptome-level gene expression measurement. However, their spatial resolution is constrained by the area and sparsity of capture domains [14]. For instance, 10x Visium uses capture spots spaced 100 μm apart [15], while traditional ST exhibits 200 μm spacing, leaving an estimated 54–80% of spatial gene expression unmeasured [16]. This substantial data sparsity reduces transcript-level spatial resolution and significantly impairs downstream analytical accuracy. Therefore, accurately inferring transcriptomic features in unmeasured spatial regions is essential for overcoming current technical limitations and fully exploiting the potential of spatial transcriptomic data.

Current approaches for predicting spatial transcriptomic gene expression can be broadly categorized into three types. Methods such as XFuse [17], Istar [18], and soScope [19] primarily employ advanced computational methods to integrate tissue information from multiple modalities, enabling the inference of super-resolution tissue structures. TCGN [20], THItoGene [21], and mclSTExp [22] focus on predicting spatial gene expression based on H&E-stained histological images. However, these two types of methods perform poorly in downstream analysis tasks, with clustering metrics such as ARI on HER2+ and CSCC datasets ranging only between 0.1–0.4. DIST [23] and stEnTrans [24] method trains a deep neural network to learn spatial dependencies in gene expression patterns across different locations, enabling interpolation and prediction for unmeasured data points. Although these methods have made notable progress in enhancing the resolution of spatial gene expression profiles, they still face challenges in accuracy and generalization for downstream tasks.

Spatial domain identification primarily includes non-spatial clustering methods and spatial clustering methods. Traditional non-spatial clustering methods, such as K-means [25], Louvain [26], and Scanpy [27], rely solely on gene expression data as input for cluster analysis, neglecting spatial location information [28,29]. To address this limitation, researchers have proposed various spatial clustering methods that integrate gene expression, spatial location, and morphological information to identify spatial domains accurately. The BayesSpace [30] models low-dimensional gene expression matrices and incorporates spatial prior to cluster truly neighboring locations. Increasingly, deep learning techniques are being adopted for spatial domain identification, as exemplified by SEDR [31], STAGATE [32], PAST [33], DeepST [34], MuCoST [35] and stHGC [36]. These approaches typically combine graph neural networks, autoencoders, and attention mechanisms to integrate spatial information and gene expression, thereby more effectively capturing spatial dependencies and complex tissue structures. Notably, the Prost [37] applies a probabilistic framework to model spatial transcriptomic data, enabling the capture of uncertainty and variability inherent in biological signals. This framework improves robustness in the presence of noise and data heterogeneity. Meanwhile, with the groundbreaking development of spatial clustering techniques, an increasing number of studies focus on integrating static spatial domain identification results with dynamic cell state transitions to construct spatiotemporally continuous biological process models [38–40]. Therefore, after identifying spatial domains in this study, we further explore the dynamic changes in cell states across different spatial domains.

To address the resolution limitations of spatial transcriptomic sequencing, we developed SpaVGN, a computational framework that integrates CNN, ViT, and GNN to predict gene expression in unsequenced tissue regions. By jointly analyzing gene expression patterns and spatial coordinates, SpaVGN enables high-fidelity transcriptomic reconstruction, simultaneously predicting unsequenced spots and integrating them with sequenced data for spatial domain identification and trajectory analysis.

## 2. Materials and methods

### 2.1. Datasets and data preprocessing

We used the Biopsy 1, Replicate 2 sample from the human melanoma dataset from Spatial Transcriptomics technology, as it covers a relatively complete spatial region of the tissue section. The tissue section is captured at a spatial resolution of 100 μm and contains 293 spatial spots and 16,148 genes. To reduce technical noise and sparsity, we apply standard filtering procedures using Scanpy, excluding genes expressed in fewer than 20 spots and removing spots with fewer than 10 detected genes. After filtering, 292 spatial spots and 11,007 genes remain.

The mouse brain dataset corresponds to the sagittal posterior section of an adult mouse brain and is generated using the 10x Genomics Visium platform. The tissue section is captured at a spatial resolution of 55 μm and contains 3,353 spatial spots and 31,053 genes in the raw count matrix. After filtering out genes detected in fewer than 10 spots and removing low-quality spots with fewer than 200 expressed genes, the dataset contains 3,339 spatial spots and 16,609 genes.

Spatial coordinates were discretized into a 2D regular grid, and gene expression values were assembled into a 3D tensor $X\in R^{G\times H\times W}$, where G is the number of genes and H×W denotes the spatial layout. For model input, the tensor was reshaped into a 4D format $X\in R^{G\times C\times H\times W}$ with C=1.

### 2.2. Overview of SpaVGN

In this study, we propose a hybrid model SpaVGN for high-quality reconstruction and completion of spatial transcriptomics data. The design of SpaVGN is motivated by the unique characteristics of spatial transcriptomics data and the limitations of existing unimodal learning approaches. ST datasets are inherently high-dimensional, spatially structured, and sparse, presenting challenges in both local signal reconstruction and global spatial understanding.

**Fig 1:**
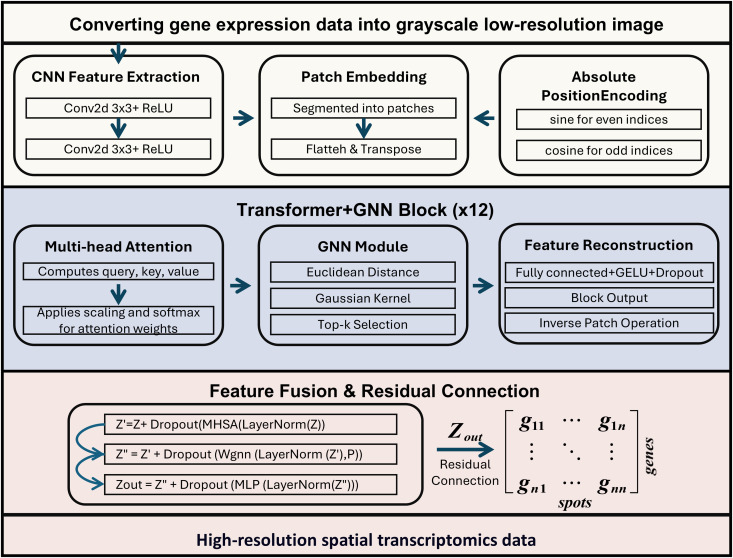
Framework for SpaVGN.

To address these challenges, SpaVGN integrates three complementary deep learning architectures: convolutional neural network (CNN), vision transformer (ViT), and graph neural network (GNN), each contributing unique capabilities. The CNN is employed to capture local spatial features and microenvironmental textures in gene expression maps. In spatial transcriptomics data, functionally related genes frequently exhibit localized co-expression patterns that the CNN can effectively model due to its localized receptive fields and translation-invariant operations. The vision transformer component is introduced to capture global contextual relationships across distant tissue regions. GNN is incorporated to explicitly encode spatial adjacency relationships between tissue spots or patches. Unlike the transformer that relies solely on learned attention weights without inherent geometric constraints, the GNN leverages biologically meaningful neighborhood graphs constructed based on either Euclidean distances or spot topology. This architectural choice ensures the model maintains both biological plausibility and spatial continuity (Fig 1).

### 2.3. Convolutional neural network module

The convolutional neural network module in SpaVGN is designed to extract localized spatial features from low-resolution gene expression maps. Spatial transcriptomics data, when arranged as two-dimensional grids, can be naturally interpreted as grayscale images with a single channel, where each pixel encodes gene expression intensity at a given spatial location. The CNN module leverages this structure to capture local gene expression patterns that are characteristic of microenvironmental variation across tissue sections.

The input to the CNN module is a single-channel tensor $X\in R^{B\times 1\times H\times W}$ where B denotes the batch size, and H, W are the spatial dimensions of the gene expression. The CNN module consists of two stacked convolutional layers with ReLU nonlinearity applied after each convolution.


$$\begin{array}{c} Z^{\left(1\right)}=Conv2d(X_{input},K^{\left(1\right)})\\ Z^{\left(2\right)}=Conv2d(Z^{\left(1\right)},K^{\left(2\right)}) \end{array}$$ (1)

The input data $X_{input}$ is received and convolved with kernel $K^{\left(1\right)}$ where $Z^{\left(1\right)}$ is the output feature map of the first convolutional layer. The second convolutional layer further processes the output of the first layer by applying convolution with kernel $K^{\left(2\right)}$*.* The CNN module functions as a front-end encoder that captures biologically relevant spatial variations in gene expression at a local level. These features are then passed to the input of the patch embedding for global context modeling.

### 2.4. Vision transformer module

To capture long-range spatial dependencies across tissue sections, we incorporate a ViT module into the SpaVGN architecture. Unlike CNN, which is limited by local receptive fields, ViT enables all-to-all patch-wise interactions through self-attention mechanisms, allowing the model to reason over global spatial context and improve expression inference in sparsely measured or biologically distant regions.

#### 2.4.1. Patch embedding and position encoding.

After CNN-based local processing, the grayscale image of gene expression is first divided into non-overlapping patches using the Patch Embedding module of the ViT. Specifically, a convolution with kernel size p×p and stride p is applied to the input feature map, transforming the original spatial structure into a series of patch vectors. After convolution, the resulting tensor has shape $R^{D\times \frac{H}{P}\times \frac{W}{p}}$, where D is the embedding dimension. Subsequently, flatten and transpose operations are applied to arrange it as:


$$Z_{0}=Flatten(Conv_{patch}(Z^{\left(2\right)}))\in R^{B\times N\times D},N=\frac{H\times W}{p^{2}}$$ (2)

where N is the number of patches.

To preserve the spatial arrangement of the non-overlapping patches extracted from the 2D gene expression maps, we incorporate absolute positional encoding into the patch embeddings. Since the self-attention mechanism in Transformers is permutation-invariant, the model must be explicitly informed of the relative and absolute positions of each patch to ensure spatial awareness.

The position encoding is defined using sine and cosine functions, with the formula as follows:


$$PE_{\left(pos,2i\right)}=\sin\left(\frac{pos}{10000^{\frac{2i}{D}}}\right),PE_{\left(pos,2i+1\right)}=\cos\left(\frac{pos}{10000^{\frac{2i}{D}}}\right)$$ (3)

where p∈[0,N−1] is the flattened patch index i is the embedding dimension index, and D is the total embedding dimension. The resulting positional encoding matrix $E_{pos}\in R^{N\times D}$ is added to the patch embedding sequence prior to self-attention computation:


$$Z_{0}^{pos}=Z_{0}+E_{pos}$$ (4)

This step allows the model to capture the spatial layout of the tissue section during global context modeling.

#### 2.4.2. Multi-Head Self-Attention.

In the context of spatial transcriptomics, MHSA enables the model to dynamically integrate expression information across distant tissue regions, capturing non-local co-expression patterns that may reflect shared functional programs or morphogenetic gradients. Unlike CNN or purely local models, the Transformer can identify context-aware dependencies even when signals are weak or noisy, a common challenge in spatial transcriptomics dataset.

In the subsequent global feature modeling, a standard Transformer module is employed. Each layer first utilizes the Multi-Head Self-Attention mechanism to model global dependencies within the input sequence, incorporating position encoding. The MHSA mechanism projects the input into queries, keys, and values:


$$Q=ZW^{Q},K=ZW^{K},V=ZW^{V}$$ (5)

where $W^{Q},W^{K},W^{V}\in R^{D\times D}$ are learnable weight matrices, then reshaped into H attention heads, each of dimension $d_{h}=D/H$, producing $Q_{h},K_{h},V_{h}\in R^{B\times H\times N\times d_{h}}$. Each head computes scaled dot-product attention:


$${Attention}_{h}(Q_{h},K_{h},V_{h})=soft\max\left(\frac{Q_{h}{K_{h}}^{T}}{\sqrt{d_{h}}}\right)V_{h}$$ (6)

Outputs from all heads are concatenated and linearly projected:


$$MASH(Z)=concat(head_{1},...,head_{H})W^{O}$$ (7)

where $W^{O}\in R^{D\times D}$ is the output projection.

### 2.5. Graph neural network module

The self-attention mechanism in the ViT module allows each patch to attend to all others in the sequence, enabling the model to capture global contextual dependencies and long-range co-expression patterns. However, this mechanism does not inherently account for the geometric proximity or topology of the underlying tissue structure. To address this limitation, we embed a GNN module into each transformer block. The GNN operates on a spatial graph constructed from the 2D coordinates of the patches, using a Gaussian kernel to define edge weights based on Euclidean distance. Through a series of localized message-passing operations, the GNN explicitly encodes spatial adjacency and reinforces topological continuity in the learned representations.

Given a sequence of N spatial patches, we assign each patch a 2D coordinate, corresponding to its row and column indices on the tissue grid. We compute a pairwise Euclidean distance matrix:


$$d_{ij}=∥p_{i}-p_{j}{∥}^{2}$$ (8)

To quantify spatial similarity, we apply a Gaussian kernel with parameter σ:


$$A_{ij}=\exp(-\frac{{d_{ij}}^{2}}{2\sigma^{2}})$$ (9)

To reduce noise interference, only the k nearest neighbors of each patch are retained (k=4 in this study). A mask matrix $M_{ij}$ is constructed where $M_{ij}=1$ if j is among the k nearest neighbors of i, otherwise $M_{ij}=0$, followed by normalization:


$${\tilde{A}}_{ij}=\frac{A_{ij}.M_{ij}}{\sum {\mathrm{\backslash nolimits}}_{j}A_{ij}.M_{ij}+\varepsilon }$$ (10)

For the input feature Z of all patches in a batch, the GNN layer aggregates the information of neighboring nodes to update each node embedding.

### 2.6. Resolution reconstruction

Following the transformer blocks, SpaVGN generates a four-channel output tensor representing four spatially offset sub-pixel predictions for each patch. This design is inspired by the sub-pixel convolution technique commonly used in image super-resolution tasks. Instead of directly regressing high-resolution results, the model predicts four interleaved subregions corresponding to a 2×2 pixel neighborhood at each low-resolution grid location.

Specifically, for an input feature map of shape $Y\in R^{B\times 4\times H\times W}$, we use k to denote the index of each of the four output channels k∈{0,1,2,3}, each of which is assigned to one of the four spatial positions in a 2×2 high-resolution patch. When k=0 represents even-row even-column positions (2i,2j); k=1 indicates even-row and odd-column (2i,2j+1); k=2 denotes odd-row and even-column (2i+1,2j); k=3 implies odd-row and odd-column (2i+1,2j+1). Where (i,j) traverse the low-resolution grid range [0,H−1]×[0,W−1], the final high-resolution output Y is constructed by interleaving these sub-pixel components to form a complete 2H×2W spatial map.

### 2.7. Loss function

The loss function in this study is based on multi-channel mean squared error with spatial masking. Through decomposition modeling and biological tissue masking mechanism, it maintains spatial topological consistency while effectively excluding interference from non-tissue regions. Let the input low-resolution image patch be $X\in R^{H\times W}$, the corresponding high-resolution target be $Y\in R^{2H\times 2W}$, and the tissue mask matrix be $M\in \{0,1{\}}^{2H\times 2W}$ (where 1 indicates valid tissue regions). The model’s predicted four-channel high-resolution output is denoted as $\hat{Y}\in R^{4\times H\times W}$, and the loss function is constructed as follows:


$$L(\hat{Y},Y,M)=\frac{1}{4N}\sum_{k=0}^{3}\sum_{i=0}^{H-1}\sum_{j=0}^{W-1}M_{i,j}^{\left(k\right)}({\hat{Y}}_{i,j}^{\left(k\right)}-Y_{i,j}^{\left(k\right)}{)}^{2}$$ (11)

In the mathematical expression of this loss function, the channel index k∈{0,1,2,3} corresponds to four sub-pixel position patterns in high-resolution space. The mask component $M_{i,j}^{\left(k\right)}$ is obtained by down-sampling the original tissue mask matrix M, defined as $M_{i,j}^{\left(k\right)}=M_{2i+[k/2],2j+(k\mathrm{mod}2)}$, which extracts binary identifiers corresponding to sub-pixel positions from the high-resolution mask. The normalization factor $N=\sum_{k=0}^{3}\sum {\mathrm{\backslash nolimits}}_{i,j}M_{i,j}^{\left(k\right)}$ dynamically counts the total number of valid tissue pixels across all channels, ensuring loss comparability between samples with different tissue morphologies.

Overall, this study proposes a hybrid CNN, ViT, and GNN model for spatial transcriptomics data reconstruction, combining CNN local feature extraction, ViT global attention modeling, and GNN spatial relationship capture. The architecture processes gene expression data through convolutional layers, patch embedding with positional encoding, multi-head self-attention, and graph-based neighborhood aggregation, followed by inverse patch reconstruction. A masked MSE loss function preserves spatial topology while excluding non-tissue regions, enabling high-resolution prediction of unmeasured gene expression patterns.

### 2.8. Performance evaluation

To assess the performance of SpaVGN in reconstructing high-resolution spatial gene expression, we designed a standardized evaluation protocol based on spatial down-sampling and reconstruction. Specifically, we simulated low-resolution inputs from full-resolution datasets and evaluated reconstruction accuracy by comparing the imputed results with the original ground truth.

#### 2.8.1. Data down-sampling and masking.

For each dataset, we first normalized the spatial coordinates to start from (0,0), and then applied grid-based down-sampling to simulate low-resolution spatial transcriptomic measurements. In the melanoma dataset, we retained spots at every second row and column position with a step size of 2, producing a uniformly down-sampled grid. For the 10x Visium dataset, which uses a honeycomb-like staggered layout, we adopted an alternating even-odd strategy to ensure accurate spatial sampling while preserving geometric patterns. In both cases, the remaining positions, which were not selected during down-sampling, were designated as masked locations, and their gene expression values were removed from the input. These masked spots served as the target locations for imputation. All imputation methods were applied to the low-resolution data to predict gene expression at these masked coordinates.

#### 2.8.2. Imputation and correlation analysis.

After imputation, the reconstructed gene expression matrices were reassembled into a full-resolution spatial grid. For SpaVGN, the predicted sub-pixel patches were rearranged into a 2 × super-resolved output and aligned with the original coordinate space. To ensure a fair comparison, only those positions within the original tissue boundary were considered in the evaluation.

We then computed gene-wise Pearson correlation coefficient between the predicted and true expression vectors across all valid spatial locations:


$$PCC=\frac{\sum_{i=1}^{N}\left(x_{i,g}-{\bar{x}}_{g})({\hat{x}}_{i,g}-{\bar{\hat{x}}}_{g}\right)}{\sqrt{\sum_{i=1}^{N}{\left(x_{i,g}-{\bar{x}}_{g}\right)}^{2}}\cdot \sqrt{\sum_{i=1}^{N}{\left({\hat{x}}_{i,g}-{\bar{\hat{x}}}_{g}\right)}^{2}}}$$ (12)

where N denotes the number of evaluated spots, $x_{i,g}$ and ${\hat{x}}_{i,g}$ are the true and predicted expression values of gene g at location i, and ${\bar{x}}_{g}$, ${\bar{\hat{x}}}_{g}$ are the corresponding means.

#### 2.8.3. Spatial clustering evaluation.

To quantitatively assess the quality of spatial domain identification produced by SpaVGN, we employed two standard unsupervised clustering metrics: the Silhouette Coefficient (SC) and the Davies–Bouldin Index (DB). The SC measures how similar each point is to its assigned cluster compared to other clusters. For a given spatial spot i with a cluster assignment label, the silhouette score is computed as:


$$sc(i)=\frac{b(i)-a(i)}{\max\{a(i),b(i)\}}$$ (13)

where a(i) is the average cluster distance between spot i and all other spots in the same predicted cluster, b(i) is the minimum average cluster distance between spot i and all spots in any other cluster. The overall SC is the average of sc(i). An SC close to 1 suggests accurate spatial domain delineation, while a value close to −1 implies possible misclassification of spots. The DB measures the average similarity between each predicted cluster and its most similar cluster, taking into account both intra-cluster compactness and inter-cluster separation. It is defined as:


$$DB=\frac{1}{K}\sum_{i=1}^{K}\underset{j\neq i}{\max}\left(\frac{S_{i}+S_{j}}{D_{ij}}\right)$$ (14)

where k is the number of predicted spatial domains, S(i) is the average distance of spots in cluster centroid, and $D_{ij}$ is the distance between the centroids of clusters i and j.

We evaluated the training and inference of SpaVGN on two benchmark datasets using a system equipped with an NVIDIA GeForce RTX 3090 GPU. On the melanoma dataset, 500 epochs plus inference required approximately 10 minutes. On the sagittal posterior mouse brain dataset, the same process took about 27 minutes.

## 3. Results

### 3.1. Algorithm evaluation of SpaVGN

**Fig 2:**
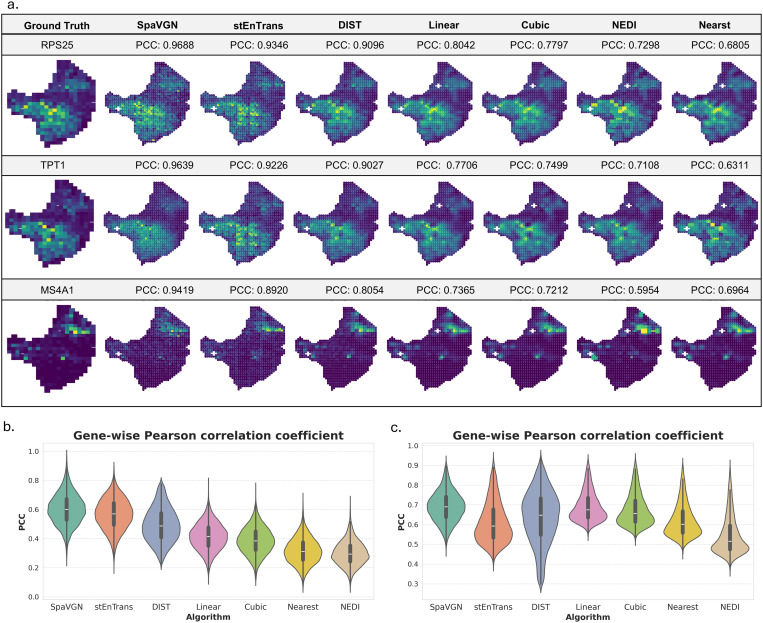
Algorithm performance comparison. (a). Pearson correlation coefficients (PCC) between predicted and true expression patterns for three representative genes (RPS25, TPT1, and MS4A1) across methods. Genes were selected from the top 3 genes with highest median PCC shared by all methods. (b). Violin plots showing gene-wise PCC distributions for all predicted genes in melanoma (left) and mouse brain (right) datasets.

To evaluate the performance of SpaVGN compared to existing methods (stEnTrans, DIST, Linear, Cubic, Nearest Neighbor and NEDI), we conducted a comprehensive analysis using melanoma and mouse brain tissue datasets. The comparison was based on Pearson correlation coefficients between predicted and true gene expression patterns. For three representative genes (RPS25, TPT1, and MS4A1), SpaVGN demonstrated higher prediction accuracy, with PCCs of 0.9688, 0.9639 and 0.9419, respectively, compared to all other methods (Fig 2a). These high-performance genes were selected from the top 3 based on median PCC rankings across all models.

Gene-wise PCC analysis further confirmed that SpaVGN maintained robust performance across the entire transcriptome (Fig 2b). In the melanoma tissue dataset, SpaVGN achieved a median PCC of 0.6090, significantly outperforming stEnTrans (0.5778), DIST (0.4968) and traditional interpolation methods Linear (0.4210), Cubic (0.3917), Nearest Neighbor (0.3187), and NEDI (0.3011). In the sagittal posterior mouse brain dataset, although all methods exhibited improved performance, SpaVGN maintained its leading position with a median PCC of 0.6816. The performance hierarchy remained consistent, with stEnTrans (0.5947), DIST (0.6293) and interpolation methods Linear (0.6674) and Cubic (0.6487) following closely, while Nearest (0.5938) and NEDI (0.5077) again demonstrated relatively lower accuracy. These results collectively demonstrate that SpaVGN provides more accurate spatial gene expression predictions across diverse tissue types and gene categories compared to existing methods.

### 3.2 SpaVGN performance in melanoma ST dataset

**Fig 3:**
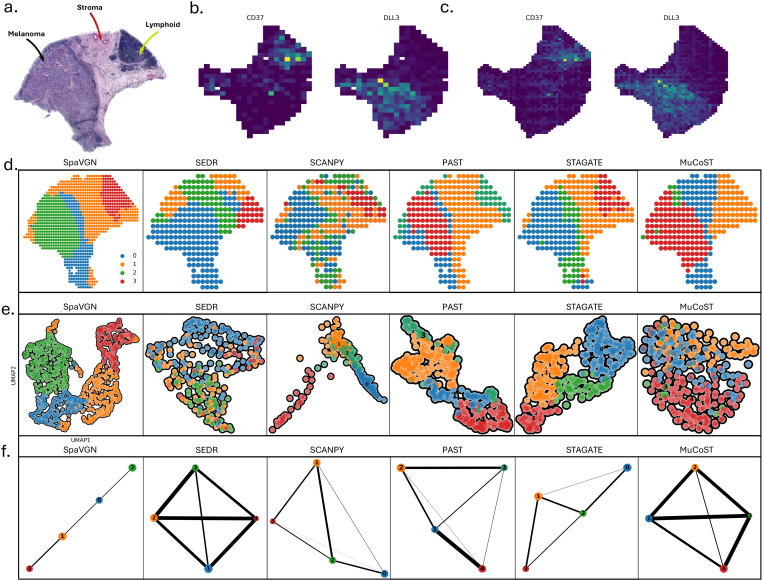
Performance evaluation of SpaVGN on melanoma ST dataset. (a). Microscopic image of melanoma tissue sections showing three main tissue types: melanoma (black arrows), stroma (red arrows), and lymphoid tissue (green arrows). Scale bar: 100 μm. (b-c). Spatial expression patterns of representative genes (CD37 and DLL3) before and after imputation. Color scale indicates normalized expression levels. (d). Performance comparison of five computational methods in tissue region segmentation. Color-coded regions correspond to different tissue domains. (e). Uniform Manifold Approximation and Projection (UMAP) visualization of cell distributions generated by five different algorithms. (f). Spatial trajectory analysis results from five methods, showing inferred developmental paths between tissue regions. Arrows indicate trajectory directions.

The histological image of melanoma tissue sections (Fig 3a) reveals three main components: melanoma, stroma, and lymphoid tissue [41]. These regions exhibit distinct morphological differences, providing an anatomical reference for subsequent analysis. To evaluate the effectiveness of gene expression imputation, we compared the spatial distribution of two representative genes, CD37 and DLL3, before and after imputation (Figs 3b–c). Prior to imputation, the expression patterns of these genes exhibited sparsity and localized absence. After imputation, the spatial continuity was significantly enhanced, and gene expression signals were noticeably restored, indicating that the imputation strategy effectively fills in missing regions and improves data completeness.

Further, we evaluated the performance of five computational methods for spatial domain identification, including SpaVGN, SEDR, SCANPY, PAST, STAGATE and MuCoST (Fig 3d). These methods exhibited varying performances in tissue region partitioning, with SpaVGN showing the highest agreement with histological annotations, indicating its ability to better preserve the spatial structural information of the tissue. To further validate the clustering consistency, we conducted UMAP-based dimensionality reduction analysis (Fig 3e). The results indicated that SpaVGN achieved clearer spatial clustering and better separation of tissue regions while maintaining biological relevance, whereas the other methods exhibited some degree of mixing between different regions. Finally, spatial trajectory inference analysis was conducted to explore the spatial relationships among different tissue regions (Fig 3f). The results demonstrated that SpaVGN and STAGATE more accurately reconstructed spatial trajectories, revealing underlying biological hierarchies, while the inferred trajectories from other methods were more scattered or lacked clear hierarchical structure.

### 3.3. SpaVGN performance in sagittal posterior mouse brain 10x visium dataset

**Fig 4:**
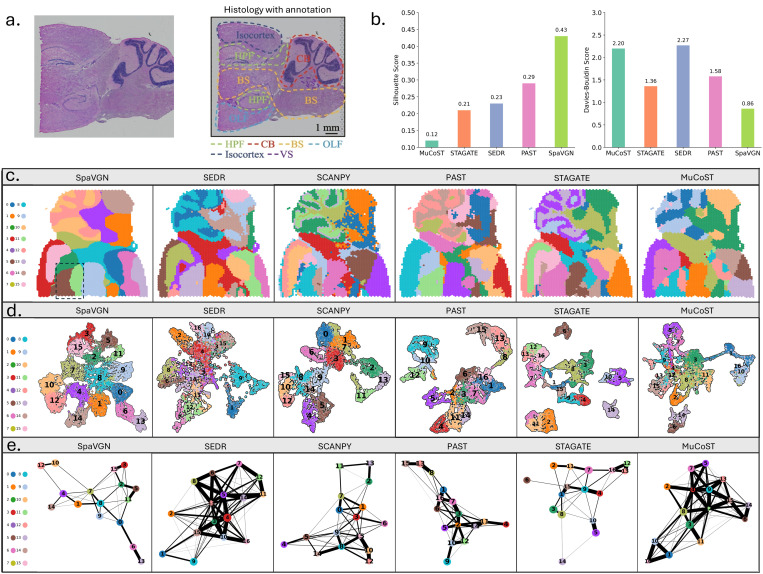
Performance evaluation of SpaVGN on Mouse Brain dataset. (a). Microscopic image of the tissue section and its annotated regions, showing the distribution of different anatomical areas. (b). Performance comparison of different methods in tissue region segmentation. The left panel shows the Separation Score, and the right panel shows the Davies-Bouldin Score. (c). Visualization of spatial domain identification results by different methods. The clustering results of each method are represented by different colors. (d). UMAP analysis results of different methods. (e). Partition-based Graph Abstraction analysis results of different methods.

This study conducted a comprehensive analysis of spatial transcriptomics data from mouse brain tissue to evaluate the performance of various algorithms in identifying spatial domains. Based on the tissue structure observed under microscopy, regions such as the Isocortex, Olfactory Bulb (OLF), Basal Ganglia (BS), Hippocampal Formation (HPF), Cerebellum (CB), and Ventral Striatum (VS) were annotated [42], providing a reference framework for subsequent analysis (Fig 4a). Due to the lack of spot-level annotations, the Silhouette Score and Davies-Bouldin Score were used to evaluate clustering performance. The results showed that SpaVGN outperformed other methods in both Silhouette Score (0.43) and Davies-Bouldin Score (0.86), compared to SEDR (SC: 0.23, DB: 2.27), PAST (SC: 0.29, DB: 1.58), STAGATE (SC: 0.21, DB: 1.36), MuCoST(SC: 0.12, DB: 2.20) indicating that SpaVGN produces more compact and well-separated clusters (Fig 4b). Due to its lack of latent variable output, Scanpy could not be evaluated using these metrics.

Furthermore, SpaVGN demonstrated high precision in identifying subregions of the Hippocampal Formation (HPF), such as the Cornu Ammonis and Dentate Gyrus [43], demonstrating its fine-grained capability in spatial domain partitioning (Fig 4c). Further analysis of the low-dimensional embeddings generated by each method showed that SpaVGN better preserves the structure of spatial domains, forming tighter and more distinct clusters (Fig 4d). The PAGA network also supported this observation, as SpaVGN constructed a clearer and sparser spatial domain connectivity graph, reflecting biologically plausible relationships between spatial domains (Fig 4e). In contrast, other methods exhibited certain limitations. The SpaVGN network accurately captured spatial relationships between cell types, with node distributions closely matching the annotated tissue regions. By comparison, SEDR and Scanpy produced noisier connectivity graphs, while PAST, STAGATE and MuCoST generated overly sparse structures that failed to fully represent the complex spatial relationships among cell types.

In UMAP analysis, SpaVGN more effectively separated distinct tissue regions, forming compact and biologically meaningful low-dimensional embedding distributions. Compared to competing methods, UMAP plots generated by SpaVGN exhibited clearer tissue domain boundaries, indicating its significant advantage in maintaining spatial structural integrity. In PAGA network analysis, SpaVGN constructed a clearer and sparser spatial domain connectivity graph that accurately reflected biologically plausible relationships between spatial domains. Its network structure demonstrated high consistency with tissue-annotated regions. In contrast, methods such as SEDR and Scanpy produced networks with noisier connections, while networks generated by PAST, STAGATE and MuCoST were overly sparse to adequately represent complex spatial relationships among cell types.

## 4. Discussion

In this study, we present SpaVGN, a hybrid deep learning framework that integrates convolutional neural networks, vision transformer, and graph neural networks to address the critical limitations of spatial transcriptomics technologies: low resolution and pervasive data sparsity. Through extensive validations on melanoma and mouse brain datasets demonstrates that SpaVGN achieves superior performance in both gene expression imputation and spatial domain identification, with notably high accuracy in transcriptomic reconstruction (melanoma median PCC up to 0.6090; mouse brain median PCC up to 0.6816).

In comparison to state-of-the-art methods such as Scanpy, SEDR, PAST, STAGATE and MuCoST, SpaVGN markedly enhances the preservation of tissue morphology and spatial continuity. Specifically, in the melanoma tissue dataset, it effectively restored missing expression signals and accurately delineated spatial domains that aligned well with histopathological annotations. In the mouse brain dataset, SpaVGN captured fine-grained structures including Cornu Ammonis and Dentate Gyrus, surpassing existing methods in terms of cluster compactness and inter-cluster separation (SC score: 0.43; DB score: 0.86). These results highlight SpaVGN’s ability to resolve subtle spatial patterns often obscured by data sparsity or noise in traditional approaches.

Furthermore, in UMAP visualizations, SpaVGN achieved clearer separation of distinct tissue regions, resulting in more compact and biologically meaningful low-dimensional embedding. Compared to other methods, the UMAP plots generated by SpaVGN exhibited clearer tissue domain boundaries, indicating its significant advantage in maintaining spatial structural integrity. Similarly, in PAGA network analysis, SpaVGN constructed a clearer and sparser spatial domain connectivity graph that accurately reflected biologically plausible relationships among spatial domains. Its network structure demonstrated high consistency with tissue-annotated regions, whereas competing approaches such as SEDR and Scanpy produced networks with noisier connections. In contrast, networks generated by PAST, STAGATE and MuCoST were excessively sparse, limiting their ability to represent the complex spatial relationships between cell types.

The success of SpaVGN can be attributed to its architectural design, which effectively captures both local microenvironmental signals and global tissue-level topological structures. The CNN module excels at extracting localized expression patterns, while the ViT component introduces global attention mechanisms to enhance contextual awareness. Furthermore, the incorporation of GNN enables SpaVGN to respect spatial adjacency of tissue spots, a critical factor for maintaining biological plausibility in downstream tasks such as spatial domain segmentation, UMAP embedding, and trajectory inference. The synergistic integration of these complementary components allows SpaVGN to model complex spatial dependencies and biological heterogeneity more comprehensively than unimodal approaches. Nevertheless, SpaVGN still has limitations requiring further investigation. For instance, while the current framework utilizes 2D spatial relationships, future work could extend SpaVGN to 3D or temporal ST datasets to model higher-dimensional tissue dynamics. Moreover, integrating H&E-stained sections or single-cell RNA-seq data may further improve resolution, interpretability, and cross-modal alignment.

In summary, SpaVGN represents a promising solution for overcoming the limitations of current spatial transcriptomics technologies. Its innovative approach and superior performance underscore its potential as a powerful tool for advancing our understanding of the intricate spatial organization and dynamics of gene expression in biological tissues.

## 5. Code availability

The code for SpaVGN can be obtained at https://github.com/BIOQM/SpaVGN/tree/master.

## Supporting information

S1 File Datasets for SpaVGN. The datasets underlying the findings of this study are available from the Figshare repository at: https://doi.org/10.6084/m9.figshare.29374538.
(PDF)
