# Supplementary material for: SpaVGN: A hybrid deep learning framework for high-resolution spatial transcriptomics data reconstruction and spatial domain identification
Source: PLoS One. 2025 Aug 14;20(8):e0329122. doi: 10.1371/journal.pone.0329122 (PMC12352682; doi:10.1371/journal.pone.0329122)
Supplement: S1 File — The datasets underlying the findings of this study are available from the Figshare repository at: https://doi.org/10.6084/m9.figshare.29374538. (PDF) [file pone.0329122.s001.pdf]

The datasets underlying the findings of this study are available from the Figshare repository at: https://doi.org/10.6084/m9.figshare.29374538.
